# Supplementary material for: Detailed molecular and epigenetic characterization of the pig IPEC-J2 and chicken SL-29 cell lines
Source: iScience. 2023 Feb 20;26(3):106252. doi: 10.1016/j.isci.2023.106252 (PMC10018572; doi:10.1016/j.isci.2023.106252)
Supplement: Data S2. Complete homer output for identified motifs in Chicken SL-29, related to Tables 5 and 6 — Homer motif analysis results for histone modifications H3K4me1, H3K4me3, H3K27ac, enhancers, and ATAC-seq of chicken SL-29 cell line. Parameters for possible false positives is as mentioned earlier for S5. [file mmc3.zip › Data_S2/S6/Chicken_SL_29/motif_analyis_enhancer_regions/knownResults.html]

motif\_analyis\_enhancer\_regions - Homer Known Motif Enrichment Results


# Homer Known Motif Enrichment Results (motif\_analyis\_enhancer\_regions)

Homer *de novo* Motif Results  
Gene Ontology Enrichment Results  
Known Motif Enrichment Results (txt file)  
Total Target Sequences = 2915, Total Background Sequences = 44110

|  |  |  |  |  |  |  |  |  |  |  |  |
| --- | --- | --- | --- | --- | --- | --- | --- | --- | --- | --- | --- |
| Rank | Motif | Name | P-value | log P-pvalue | q-value (Benjamini) | # Target Sequences with Motif | % of Targets Sequences with Motif | # Background Sequences with Motif | % of Background Sequences with Motif | Motif File | SVG |
| 1 | T G C A A C T G T A C G A T G C A G T C G A C T T C G A A T C G | ZNF711(Zf)/SHSY5Y-ZNF711-ChIP-Seq(GSE20673)/Homer | 1e-36 | -8.299e+01 | 0.0000 | 1378.0 | 47.27% | 15813.4 | 35.85% | motif file (matrix) | svg |
| 2 | C T G A A C T G A C T G A G T C A G T C A G C T C T A G T A C G | ZFX(Zf)/mES-Zfx-ChIP-Seq(GSE11431)/Homer | 1e-25 | -5.923e+01 | 0.0000 | 680.0 | 23.33% | 6956.6 | 15.77% | motif file (matrix) | svg |
| 3 | G A T C C T G A A G T C C G A T C G A T G A T C A G T C A C T G A T C G A G C T | Elk4(ETS)/Hela-Elk4-ChIP-Seq(GSE31477)/Homer | 1e-23 | -5.359e+01 | 0.0000 | 535.0 | 18.35% | 5254.5 | 11.91% | motif file (matrix) | svg |
| 4 | G A T C T C G A A G T C C G A T C G A T A G T C A T G C A C T G A T C G G A C T | Elk1(ETS)/Hela-Elk1-ChIP-Seq(GSE31477)/Homer | 1e-19 | -4.482e+01 | 0.0000 | 542.0 | 18.59% | 5571.4 | 12.63% | motif file (matrix) | svg |
| 5 | C T G A T G C A T A G C T G A C T A C G T C A G C T G A G C T A T C A G G A C T | ELF1(ETS)/Jurkat-ELF1-ChIP-Seq(SRA014231)/Homer | 1e-17 | -4.122e+01 | 0.0000 | 459.0 | 15.75% | 4614.0 | 10.46% | motif file (matrix) | svg |
| 6 | C T G A T A G C T G A C T C A G C T A G G T C A C G T A T C A G A G C T T C A G | ETV4(ETS)/HepG2-ETV4-ChIP-Seq(ENCODE)/Homer | 1e-15 | -3.490e+01 | 0.0000 | 759.0 | 26.04% | 8783.1 | 19.91% | motif file (matrix) | svg |
| 7 | T A G C C G T A C T G A T A C G C G T A A C G T A C T G A C T G A G T C T A C G C T A G G T A C | YY1(Zf)/Promoter/Homer | 1e-13 | -2.995e+01 | 0.0000 | 175.0 | 6.00% | 1452.4 | 3.29% | motif file (matrix) | svg |
| 8 | A G T C C T G A A G T C C G A T C A G T G A T C A T G C A C T G A T C G G A C T | Fli1(ETS)/CD8-FLI-ChIP-Seq(GSE20898)/Homer | 1e-12 | -2.919e+01 | 0.0000 | 650.0 | 22.30% | 7520.9 | 17.05% | motif file (matrix) | svg |
| 9 | T C G A T C G A T A G C G T A C T C A G T A C G C G T A C G T A T C A G A G C T | GABPA(ETS)/Jurkat-GABPa-ChIP-Seq(GSE17954)/Homer | 1e-12 | -2.912e+01 | 0.0000 | 456.0 | 15.64% | 4933.8 | 11.19% | motif file (matrix) | svg |
| 10 | A T C G A G T C A G T C G A C T A T G C C T G A C T A G A C T G T A C G G T A C C T G A C G A T | AP-2gamma(AP2)/MCF7-TFAP2C-ChIP-Seq(GSE21234)/Homer | 1e-12 | -2.795e+01 | 0.0000 | 1223.0 | 41.96% | 15698.3 | 35.59% | motif file (matrix) | svg |
| 11 | T G C A T C G A T A G C G T A C T C A G C T A G G T C A G C T A T C A G G A C T | ETS(ETS)/Promoter/Homer | 1e-11 | -2.613e+01 | 0.0000 | 257.0 | 8.82% | 2497.2 | 5.66% | motif file (matrix) | svg |
| 12 | T C G A T C G A T A C G G A T C G T C A G T A C C G A T A G C T G T C A T G C A | Nkx3.1(Homeobox)/LNCaP-Nkx3.1-ChIP-Seq(GSE28264)/Homer | 1e-11 | -2.582e+01 | 0.0000 | 523.0 | 17.94% | 5938.4 | 13.46% | motif file (matrix) | svg |
| 13 | T C G A C T G A T A G C T G A C T C A G T C A G C G T A C G T A T C A G A G C T | ETV1(ETS)/GIST48-ETV1-ChIP-Seq(GSE22441)/Homer | 1e-10 | -2.309e+01 | 0.0000 | 590.0 | 20.24% | 6958.4 | 15.78% | motif file (matrix) | svg |
| 14 | C T G A A G T C C G A T A G C T A T G C G T A C A C G T A T C G C A G T G C A T | Elf4(ETS)/BMDM-Elf4-ChIP-Seq(GSE88699)/Homer | 1e-9 | -2.292e+01 | 0.0000 | 379.0 | 13.00% | 4139.2 | 9.38% | motif file (matrix) | svg |
| 15 | G C T A C G A T C A T G A T G C A G T C A G T C G A C T T A C G T C G A C T A G A C T G T A G C | AP-2alpha(AP2)/Hela-AP2alpha-ChIP-Seq(GSE31477)/Homer | 1e-9 | -2.178e+01 | 0.0000 | 955.0 | 32.76% | 12153.8 | 27.55% | motif file (matrix) | svg |
| 16 | G A C T T C G A C G A T T A C G C T A G T A C G A C T G A T G C G T A C G T A C | Zac1(Zf)/Neuro2A-Plagl1-ChIP-Seq(GSE75942)/Homer | 1e-8 | -1.944e+01 | 0.0000 | 1502.0 | 51.53% | 20360.3 | 46.16% | motif file (matrix) | svg |
| 17 | C T G A A T G C C G T A A C G T A G T C A G T C A C G T A C T G A T C G G C A T | SPDEF(ETS)/VCaP-SPDEF-ChIP-Seq(SRA014231)/Homer | 1e-8 | -1.864e+01 | 0.0000 | 296.0 | 10.15% | 3209.8 | 7.28% | motif file (matrix) | svg |
| 18 | C G T A T A G C T A G C T G C A A C T G C T A G C G T A C G T A T C A G G A C T | EHF(ETS)/LoVo-EHF-ChIP-Seq(GSE49402)/Homer | 1e-6 | -1.424e+01 | 0.0000 | 350.0 | 12.01% | 4097.9 | 9.29% | motif file (matrix) | svg |
| 19 | C T G A T C A G G T A C G C T A A C T G T G A C G C A T C A T G | SCL(bHLH)/HPC7-Scl-ChIP-Seq(GSE13511)/Homer | 1e-5 | -1.256e+01 | 0.0001 | 1584.0 | 54.34% | 22127.1 | 50.16% | motif file (matrix) | svg |
| 20 | G C T A A G T C T A C G T G C A A T C G T C A G G C T A T C G A T C A G A G C T | ELF5(ETS)/T47D-ELF5-ChIP-Seq(GSE30407)/Homer | 1e-5 | -1.255e+01 | 0.0001 | 211.0 | 7.24% | 2324.4 | 5.27% | motif file (matrix) | svg |
| 21 | T C G A T A G C T G C A A C T G A C T G C G T A C G T A C T A G G A C T T A C G | ETS1(ETS)/Jurkat-ETS1-ChIP-Seq(GSE17954)/Homer | 1e-5 | -1.252e+01 | 0.0001 | 464.0 | 15.92% | 5746.4 | 13.03% | motif file (matrix) | svg |
| 22 | T C G A T A G C G T C A A C T G A C T G C G T A C G T A C T A G A G C T T C A G | ERG(ETS)/VCaP-ERG-ChIP-Seq(GSE14097)/Homer | 1e-5 | -1.229e+01 | 0.0001 | 523.0 | 17.94% | 6583.4 | 14.93% | motif file (matrix) | svg |
| 23 | T A G C C G A T T A C G A C T G A G T C A C T G A T C G A T C G C G T A C T G A | E2F1(E2F)/Hela-E2F1-ChIP-Seq(GSE22478)/Homer | 1e-4 | -1.128e+01 | 0.0002 | 535.0 | 18.35% | 6816.6 | 15.45% | motif file (matrix) | svg |
| 24 | A T G C A G T C C T G A A G T C C G A T A C G T A G T C A G T C A C G T A T C G G A C T A C G T | Etv2(ETS)/ES-ER71-ChIP-Seq(GSE59402)/Homer | 1e-4 | -1.062e+01 | 0.0004 | 330.0 | 11.32% | 4002.0 | 9.07% | motif file (matrix) | svg |
| 25 | T G C A C T G A A G T C G T C A A C T G A C T G C G T A C G T A C T G A A G C T | EWS:FLI1-fusion(ETS)/SK\_N\_MC-EWS:FLI1-ChIP-Seq(SRA014231)/Homer | 1e-4 | -9.581e+00 | 0.0012 | 226.0 | 7.75% | 2644.8 | 6.00% | motif file (matrix) | svg |
| 26 | T A C G T A C G G T A C A T C G T A C G T A C G G T C A C T G A C G T A G A C T | E2F4(E2F)/K562-E2F4-ChIP-Seq(GSE31477)/Homer | 1e-4 | -9.373e+00 | 0.0014 | 752.0 | 25.80% | 10065.5 | 22.82% | motif file (matrix) | svg |
| 27 | T A C G T A C G G T A C A T C G A C T G T A C G T C G A C T G A T C G A A T C G | E2F6(E2F)/Hela-E2F6-ChIP-Seq(GSE31477)/Homer | 1e-3 | -8.500e+00 | 0.0033 | 1041.0 | 35.71% | 14381.3 | 32.60% | motif file (matrix) | svg |
| 28 | T A C G G A C T A C T G A C T G C T A G A T G C A G T C A G T C A G T C C T G A | ZNF692(Zf)/HEK293-ZNF692.GFP-ChIP-Seq(GSE58341)/Homer | 1e-3 | -7.952e+00 | 0.0055 | 107.0 | 3.67% | 1147.5 | 2.60% | motif file (matrix) | svg |
| 29 | C T A G C T A G A G T C T C A G A C T G A C G T A C G T C T G A | MYB(HTH)/ERMYB-Myb-ChIPSeq(GSE22095)/Homer | 1e-3 | -7.485e+00 | 0.0085 | 753.0 | 25.83% | 10250.6 | 23.24% | motif file (matrix) | svg |
| 30 | A T G C T C G A T A C G A C G T A T G C A G T C A C G T A G T C A G T C G A T C | Znf263(Zf)/K562-Znf263-ChIP-Seq(GSE31477)/Homer | 1e-3 | -6.914e+00 | 0.0146 | 1262.0 | 43.29% | 17845.5 | 40.46% | motif file (matrix) | svg |
| 31 | C G A T C T A G A C G T A C G T A C G T C G T A A G C T C G A T A G C T C G T A C T A G T A G C | FoxD3(forkhead)/ZebrafishEmbryo-Foxd3.biotin-ChIP-seq(GSE106676)/Homer | 1e-2 | -6.907e+00 | 0.0146 | 63.0 | 2.16% | 627.1 | 1.42% | motif file (matrix) | svg |
| 32 | C A G T T C A G A G C T G A C T A C G T A G T C G A T C G A C T C T G A A C T G G A T C C G T A C T G A A G T C G T A C | Rfx6(HTH)/Min6b1-Rfx6.HA-ChIP-Seq(GSE62844)/Homer | 1e-2 | -6.176e+00 | 0.0286 | 509.0 | 17.46% | 6835.4 | 15.50% | motif file (matrix) | svg |
| 33 | C G T A T G A C T A G C T G C A A C T G A C T G C G T A C G T A T C A G G A C T | ELF3(ETS)/PDAC-ELF3-ChIP-Seq(GSE64557)/Homer | 1e-2 | -6.145e+00 | 0.0286 | 143.0 | 4.91% | 1692.1 | 3.84% | motif file (matrix) | svg |
| 34 | A G T C G T A C A G C T C T A G A G T C C G A T A C T G C G T A A C T G G T C A | Zic(Zf)/Cerebellum-ZIC1.2-ChIP-Seq(GSE60731)/Homer | 1e-2 | -5.549e+00 | 0.0504 | 564.0 | 19.35% | 7692.4 | 17.44% | motif file (matrix) | svg |
| 35 | T C G A T G C A A C T G G T C A A G T C A G T C A G T C A G T C A G C T T G A C | LRF(Zf)/Erythroblasts-ZBTB7A-ChIP-Seq(GSE74977)/Homer | 1e-2 | -5.240e+00 | 0.0666 | 1319.0 | 45.25% | 18916.8 | 42.89% | motif file (matrix) | svg |
| 36 | A C T G A C G T C A T G A T C G A T C G T G A C A C T G A T C G A T C G T G C A C T G A C G T A | E2F3(E2F)/MEF-E2F3-ChIP-Seq(GSE71376)/Homer | 1e-2 | -4.851e+00 | 0.0955 | 1065.0 | 36.54% | 15166.5 | 34.38% | motif file (matrix) | svg |
| 37 | T C A G G C T A T C A G C A G T T G A C G T C A A G T C A T C G T G C A G T A C C A G T G A T C | Npas4(bHLH)/Neuron-Npas4-ChIP-Seq(GSE127793)/Homer | 1e-2 | -4.799e+00 | 0.0979 | 583.0 | 20.00% | 8050.3 | 18.25% | motif file (matrix) | svg |
| 38 | A G T C C T G A A T C G A G C T A G C T G A C T A G T C G C T A A C G T C G A T G C A T C G A T A T C G C G T A T A G C G C A T A T G C C G T A | bZIP:IRF(bZIP,IRF)/Th17-BatF-ChIP-Seq(GSE39756)/Homer | 1e-2 | -4.747e+00 | 0.1005 | 22.0 | 0.75% | 188.0 | 0.43% | motif file (matrix) | svg |
| 39 | A G T C A C G T A C T G A G C T A C G T A C G T G T C A A G T C | Foxo1(Forkhead)/RAW-Foxo1-ChIP-Seq(Fan\_et\_al.)/Homer | 1e-2 | -4.720e+00 | 0.1005 | 288.0 | 9.88% | 3798.8 | 8.61% | motif file (matrix) | svg |
| 40 | T G A C G T C A A G T C G C T A C T A G A G T C C T G A C A T G A C T G C T A G T A C G T C A G | Zic2(Zf)/ESC-Zic2-ChIP-Seq(SRP197560)/Homer | 1e-2 | -4.694e+00 | 0.1006 | 754.0 | 25.87% | 10573.5 | 23.97% | motif file (matrix) | svg |
| 41 | A G C T A G C T T A G C A T C G A G T C A C T G A T G C A T C G T C G A C T G A T C G A C T G A | E2F(E2F)/Hela-CellCycle-Expression/Homer | 1e-2 | -4.678e+00 | 0.1006 | 59.0 | 2.02% | 644.1 | 1.46% | motif file (matrix) | svg |
| 42 | G A C T C T A G C T A G A G T C T G C A A C T G A C G T A C G T C T A G T C A G | AMYB(HTH)/Testes-AMYB-ChIP-Seq(GSE44588)/Homer | 1e-2 | -4.655e+00 | 0.1006 | 484.0 | 16.60% | 6624.3 | 15.02% | motif file (matrix) | svg |
